# The redox-sensitive protein HMGB1: intracellular and extracellular roles

**DOI:** 10.1038/s12276-026-01640-3

**Published:** 2026-02-13

**Authors:** Man Sup Kwak, Su Ful Jung, In Ho Park, Jeon-Soo Shin

**Affiliations:** 1 Department of Microbiology, Yonsei University College of Medicine, Seoul, South Korea; 2 Institute for Immunology and Immunological Diseases, Yonsei University College of Medicine, Seoul, South Korea; 3 Brain Korea 21 FOUR Project for Medical Science, Yonsei University College of Medicine, Seoul, South Korea; 4 Department of Biomedical Science, Yonsei University College of Medicine, Seoul, South Korea

**Keywords:** Inflammation, Innate immunity

## Abstract

HMGB1 is a non-histone nuclear protein that is primarily located in the nucleus. It can be translocated to the cytoplasm and secreted into the extracellular space upon stimulation. In the nucleus, HMGB1 functions as a DNA chaperone; in the cytoplasm, it participates in autophagy and mitochondrial homeostasis; and in the extracellular environment, it acts as a damage-associated molecular pattern. HMGB1 consists of three cysteine residues (C^23^, C^45^ and C^106^) and is a redox-sensitive protein that exists in distinct redox isoforms: reduced (Re-HMGB1), disulfide (Ds-HMGB1), oxidized (Ox-HMGB1) and dimerized (Di-HMGB1). The localization-specific functions of HMGB1 are regulated by its redox state, which is involved in preventing DNA damage, inflammation, cell death and survival, and various inflammatory disorders. This Review describes the oxidation mechanisms of HMGB1 and summarizes its functional roles in the nucleus, cytoplasm and extracellular space depending on its redox status. We further describe the oxidation states of HMGB1 released during different forms of cell death, the distinct redox isoform of HMGB1 and its possible association with various diseases. This Review provides insights into therapeutic strategies targeting redox-sensitive damage-associated molecular patterns in inflammatory and autoimmune pathologies.

## HMGB1 redox states influence cell death and inflammation

This Review explores the role of HMGB1, a protein involved in immune responses and inflammation. HMGB1 can exist in different forms depending on its oxidation state, which affects its function. The study addresses how these forms influence inflammation and disease. Researchers found that HMGB1 can act as a danger signal (damage-associated molecular pattern) when released from cells, triggering immune responses. They outline HMGB1’s behavior under different conditions, focusing on its redox states. These states include reduced (Re-HMGB1), disulfide (Ds-HMGB1) and oxidized (Ox-HMGB1) forms, each playing distinct roles in inflammation and cell death. Ds-HMGB1 is linked to chronic inflammation and diseases such as sepsis and cancer. By contrast, Re-HMGB1 helps with tissue repair. In addition, a dimerized form (Di-HMGB1) was recently identified, showing enhanced immune-boosting activity. Understanding these roles could lead to new treatments for inflammatory diseases.

This summary was initially drafted using artificial intelligence, then revised and fact-checked by the author.

## Introduction

**Fig. 1:**
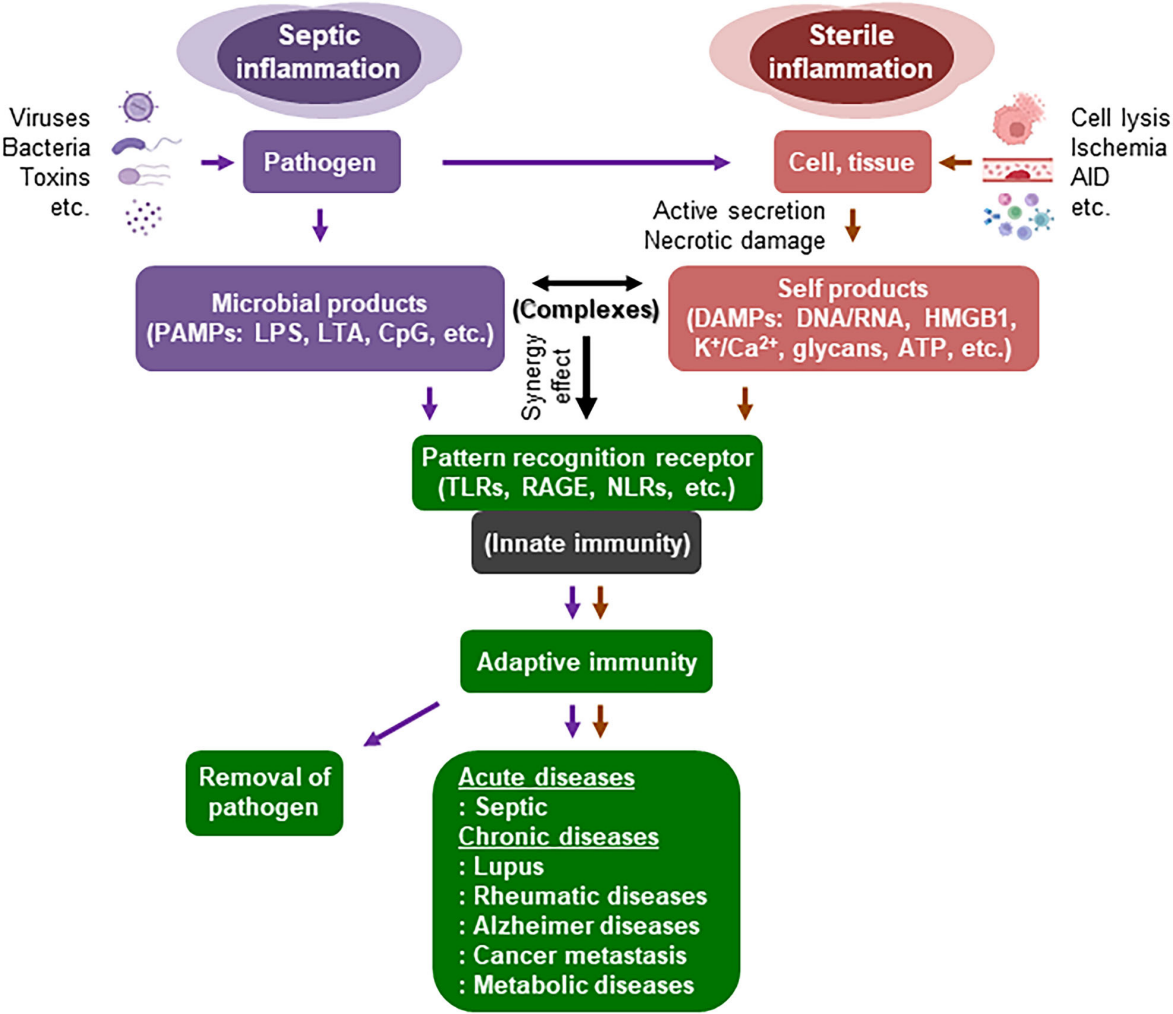
Distinct and overlapping pathways in septic and sterile inflammation. Septic inflammation is initiated by pathogens such as viruses, bacteria or microbial toxins, leading to the release of PAMPs (LPS, LTA, and CpG). By contrast, sterile inflammation results from tissue or cellular damage caused by ischemia or AID, leading to the active secretion or necrotic release of self-derived DAMPs (HMGB1, HSPs, S100, Prx, and Trx). Both PAMPs and DAMPs activate pattern recognition receptors (PRRs) such as TLRs and RAGE, initiating innate immune responses. These signals act independently, synergistically or as molecular complexes to enhance immune activation. Subsequent adaptive immune responses contribute to pathogen clearance; however, if unresolved, they may lead to disease progression. While septic inflammation is primarily linked to infection-related pathologies, sterile inflammation also regulates intracellular signaling and contributes to tissue homeostasis. AID, autoimmune diseases.

Damage-associated molecular patterns (DAMPs) are endogenous danger signals generated in response to pathogen invasion, cellular damage or oxidative stress. Under normal physiological conditions, DAMPs do not trigger immune responses. However, excessive release of DAMPs, or changes in their biochemical or structural properties, can activate the immune system by engaging innate immune receptors and initiating inflammation. DAMPs are classified on the basis of their molecular characteristics as follows: (1) nucleic acids, including both intracellular and extracellular DNA and RNA, as well as mitochondrial DNA; (2) cellular proteins, including HMGB1, heat shock proteins (HSPs), S100 proteins, thioredoxin (Trx), peroxiredoxins (Prxs) and cytokines such as interleukin (IL)-1α and IL-1β; (3) ions, including potassium (K⁺) and calcium (Ca²⁺), which are recognized as ionic DAMPs; (4) glycans, including hyaluronan and decorin, which are key representatives of carbohydrate DAMPs; and (5) metabolites, including adenosine triphosphate (ATP), uric acid and succinate^1–4^. Pathogen-associated molecular pattern (PAMPs) are conserved molecular structures uniquely expressed by bacteria, viruses and other infectious microorganisms. These pathogen-specific motifs are recognized by a host’s innate immune system, facilitating rapid and efficient detection of invading pathogens. Representative PAMPs include lipopolysaccharide (LPS), lipoteichoic acid (LTA), peptidoglycan, viral double-stranded RNA and single-stranded RNA, β-glucan, mannans, flagellin and unmethylated cytosine-phosphate-guanine (CpG) DNA motifs^5^. Beyond these categories, certain DAMPs can form complexes with PAMPs that potentiate immune responses. For example, HMGB1–LPS or HMGB1–LTA complexes can be formed, significantly increasing inflammatory signaling^6^. DAMPs and PAMPs are sensed by a variety of pattern recognition receptors that are expressed in both immune and non-immune cells. Intracellular sensing of DAMPs activates cell-intrinsic sterile inflammatory responses, which may facilitate cellular repair but can become pathogenic if persistently activated^7^. Major DAMP- and PAMP-sensing receptors include Toll-like receptors (TLRs), receptor for advanced glycation endproducts (RAGE), nucleotide-binding oligomerization domain-like receptors (NLRs), retinoic acid-inducible gene I-like receptors (RLRs) and cyclic GMP–AMP synthase-stimulator of interferon genes (cGAS-STING). Activation of these receptors leads to the production of inflammatory cytokines and chemokines, the recruitment of immune cells and the amplification of the inflammatory response^1,4^. DAMPs and their receptors play central roles in the pathogenesis of various diseases^7^. By excessively activating immune responses, DAMPs contribute to autoimmune diseases such as rheumatoid arthritis and systemic lupus erythematosus^8,9^. DAMPS are also involved in metabolic disorders, including type 2 diabetes and obesity^2,10^, as well as neurodegenerative conditions, such as Alzheimer’s disease^11^. In the tumor microenvironment (TME), DAMPs can modulate immune activity and influence cancer progression and metastasis^12^ (Fig. 1). Understanding the multifaceted roles of DAMPs provides valuable insights into immune regulation and highlights potential therapeutic targets for a range of inflammatory and immune-mediated diseases.

HMG proteins are non-histone chromatin-binding proteins that are characterized by their rapid mobility during electrophoresis and their ability to bind to DNA. There are three major subfamilies of these proteins: HMGA, HMGB and HMGN. Each subfamily has distinct structural motifs, nuclear localization patterns and biological functions^13,14^. HMGA proteins contain three AT-hook DNA-binding domains that bind preferentially to AT-rich regions of DNA. They influence gene regulation during development and differentiation by modulating chromatin architecture. HMGB proteins consist of two HMG-box domains (box A and box B) and an acidic C-terminal containing a string of glutamic and aspartic acid residues. HMGN proteins contain a nucleosome-binding domain that allows them to bind specifically to nucleosomes. This interaction modulates histone structure and enhances the accessibility of transcription factors, thereby promoting transcriptional activity^13,14^.

HMGB1 is a ubiquitously expressed, non-histone nuclear protein that is abundantly expressed in almost all cell types that contain a nucleus. First discovered in 1973 in calf thymus tissue, HMGB1 was identified as a nuclear protein involved in chromatin architecture^15^. In 1999, Wang et al. demonstrated its immunological significance when they showed that HMGB1 acts as a proinflammatory mediator in LPS-mediated endotoxemia^16^. HMGB1 is released during necrotic cell death and promotes innate immune responses^17^. Over the past 26 years, extensive research has been conducted to identify the role of HMGB1 in inflammation, its secretion mechanism, the interaction with endogenous host molecules and PAMPs, and intracellular function. HMGB1 is a redox-sensitive protein, and its immune functions vary depending on its oxidation state. This Review aims to investigate HMGB1’s oxidation mechanisms and elucidate its roles and functions under different oxidation states from immunological and pathological perspectives.

## Reactive oxygen species (ROS) generation in inflammation and ROS-sensitive DAMPs

Inflammatory conditions are closely associated with increased intracellular ROS production, which plays a pivotal role in modulating immune responses and contributing to tissue damage. During inflammation, immune cells such as neutrophils and macrophages undergo a ‘respiratory burst’, which leads to a significant increase in ROS production. This process is primarily mediated by the activation of nicotinamide adenine dinucleotide phosphate hydrogen (NADPH) oxidase complexes, particularly NADPH oxidase 2 (NOX2), which generate superoxide anions and other ROS^18^. Mitochondria are another major source of ROS during inflammatory responses. Inflammatory stimuli can disrupt mitochondrial electron transport chains, leading to electron leakage and subsequent ROS generation. These ROS further amplify inflammatory signaling pathways, including nuclear factor kappa-light-chain-enhancer of activated B cells (NF-κB), nod-like receptor family pyrin domain-containing 3 (NLRP3) inflammasome, mitogen-activated protein kinase (MAPK), and Janus kinase 2 (JAK2)–signal transducer and activator of transcription 3 (STAT3) pathways^19,20^. Excessive ROS production can overwhelm cellular antioxidant defenses, resulting in oxidative stress. This condition can damage proteins, lipids and DNA, contributing to cell dysfunction and death and exacerbating inflammatory conditions^20^.

During inflammation, increased intracellular ROS can oxidize proteins involved in immune signaling. These redox modifications can modulate immune responses and contribute to disease progression. For example, ROS can increase IL-8 expression, thereby enhancing its chemotactic activity toward neutrophils. Antioxidants such as *N*-acetylcysteine can suppress IL-8 production, suggesting redox-sensitive regulation^21^. In rhabdomyosarcoma cells, inhibition of nuclear receptor 4A1 leads to the accumulation of ROS, which subsequently induces IL-24 expression in a ROS-dependent manner, thereby suppressing cellular proliferation, survival and migration^22^. Oxidation also leads to the formation of malondialdehyde (MDA) adducts on proteins, generating neo-epitopes that can trigger autoimmune responses, as seen in rheumatoid arthritis. Elevated levels of antibodies against MDA-modified proteins correlate with disease activity in autoimmune conditions^23^. Furthermore, ROS levels influence macrophage polarization: high ROS promote the proinflammatory M1 phenotype, while antioxidants promote the anti-inflammatory M2 phenotype^24^. HMGB1 is also a redox-sensitive protein that influences various inflammatory functions depending on its oxidation state^14^.

## HMGB1 structure

**Fig. 2:**
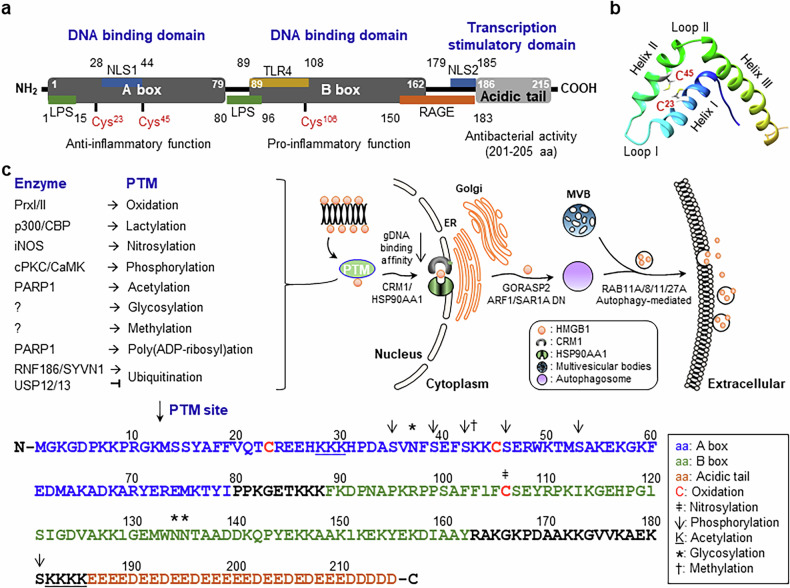
Structure of HMGB1 and PTMs of HMGB1. **a**, Schematic structure of HMGB1. HMGB1 consists of two DNA-binding domains (A box and B box) and C-terminal acidic tail with transcriptional activity. The A box includes Cys^23^ and Cys^45^ forming intramolecular disulfide bonds, linked to anti-inflammatory function. The B box contains redox-sensitive Cys^106^, critical for proinflammatory signaling. The acidic tail is associated with antibacterial activity. Two NLS regions (NLS1: a.a. 28–44, NLS2: a.a. 179–185) facilitate nuclear import. Key binding sites for LPS, TLR4 and RAGE are indicated. **b**, Model structure of HMGB1 A box. The structural model (PDB ID: 4QR9, 2MSY, 2RTU and 8I9M) was generated using the Modeller 10.7 program. The A box comprises three α-helices (I–III) and two loops (I and II). Cys^23^ and Cys^45^ are spatially close, capable of intramolecular disulfide bond formation in response to ROS. **c**, Enzymes and specific PTM sites of HMGB1. Multiple enzymes induce distinct PTMs of HMGB1: oxidation, lactylation, nitrosylation, phosphorylation, acetylation, glycosylation, methylation, poly(ADP-ribosyl)ation, ubiquitination and deubiquitination. PTMs modulate HMGB1 subcellular localization, genomic DNA (gDNA)-binding affinity and secretion via ER–Golgi, MVBs or autophagy. Key proteins involved in HMGB1 trafficking include CRM1/HSP90AA1, GORASP2, ARF/SAR1A, and Rab11A/8/11/27A. The full-length HMGB1 sequence is shown with annotated domains: A box (blue), B box (green) and acidic C tail (orange). PTM sites are indicated: oxidation (red C), nitrosylation (‡), phosphorylation (↓), acetylation (K), glycosylation (*) and methylation (†). Domain-specific PTMs regulate HMGB1 structure, localization and function. ER, endoplasmic reticulum; MVBs, multivesicular bodies; iNOS, inducible NO synthase.

HMGB1 is composed of 215 amino acids organized into two domains: the A box (amino acids (a.a.) 1–79) and the B box (a.a. 89–162). These domains exhibit anti- and proinflammatory properties, respectively. The B box domain activates immune cells via TLR4 and RAGE receptors^25^. The acidic tail (a.a. 186–215) is rich in acidic residues that regulate the DNA-binding activity of the A/B boxes and facilitate transcriptional stimulation by interacting with transcriptional cofactors. Notably, a short peptide (a.a. 201–205) in this region exhibits antimicrobial activity^14,26^. The A and B boxes bind to the minor groove of DNA and induce bending and unwinding of the DNA in the nucleus, acting as DNA chaperones^27^. This function facilitates transcription regulation, replication, recombination and DNA repair^13,14,28^. HMGB1 plays critical roles in both intracellular chromatin architecture and extracellular inflammatory signaling when secreted. It has two nuclear localization signals (NLS1, a.a. 28–44; NLS2, a.a. 179–185) that are essential for the nuclear import of newly translated HMGB1. The segment a.a. 89–108 is crucial for binding to TLR4, which facilitates innate immune activation and cytokine production in response to tissue damage or infection^25^. The C-terminal region of B box (a.a. 150–183) constitutes the binding site for RAGE. The HMGB1–RAGE interaction promotes cell migration, survival and sustained inflammatory responses, contributing to chronic inflammation, tumor progression and metastasis^29^. The A box domain (a.a. 1–15) and overlapping regions within the B box (a.a. 80–96) interact with bacterial LPS and transfer it to CD14^30^. These HMGB1-derived peptides can bind to distinct domains of LPS and have been shown to inhibit LPS-induced inflammatory responses in murine endotoxemia models. This suggests a potential anti-inflammatory therapeutic application^31^ (Fig. 2a).

The redox-sensitive protein HMGB1 contains three cysteine residues (C^23^, C^45^ and C^106^) and four distinct redox isoforms: reduced (Re)-HMGB1, disulfide (Ds)-HMGB1, oxidized (Ox)-HMGB1 and dimeric (Di)-HMGB1. The following sections focus on the localization-specific functions of the different HMGB1 redox forms in DNA damage, inflammation, cell death and survival, and various inflammatory disorders. The structure of the HMGB1 A box was modeled using the Modeller 10.7 program based on the structural templates from the Protein Data Bank (PDB IDs: 4QR9, 2MSY, 2RTU and 8I9M). The HMGB1 A box comprises three α-helices (helices I, II and III) and two loops (loops I and II). Two cysteine residues (Cys^23^ and Cys^45^) are structurally proximal and capable of forming an intramolecular disulfide bond in response to ROS (Fig. 2b).

HMGB1 secretion and activity are tightly regulated by various post-translational modifications (PTMs), which modulate its DNA-binding ability, intracellular localization and secretion mechanisms. In response to ROS, PrxI and PrxII are oxidized, which activates their sensor function and transmits oxidative signals to HMGB1. HMGB1 oxidation leads to the formation of a disulfide bond between its Cys^23^ and Cys^45^ residues. This results in a conformational change that facilitates nucleocytoplasmic translocation^32^. Under inflammatory conditions or in high-glucose environments, lactate levels within cells increase, activating the histone acetyltransferases p300/CBP. These enzymes catalyze the lactylation of HMGB1, enhancing its cytoplasmic accumulation and secretion^33^. Inducible nitric oxide (NO) synthase is activated during inflammation and generates NO, which induces S-nitrosylation of HMGB1 at Cys^106^ in the brain microglia and macrophages. This HMGB1 modification facilitates its secretion into the extracellular space, promoting proinflammatory and neurodegenerative effects^34^. Classical protein kinase C (cPKC) plays a critical role in inflammatory signaling by phosphorylating multiple serine residues (Ser^35,39,42,46,53,181^) in HMGB1^35–37^. Calcium/calmodulin-dependent protein kinase (CaMK) IV also induces HMGB1 phosphorylation. This phosphorylation weakens the nuclear retention signal of HMGB1, promoting its translocation to the cytoplasm^38^. Acetylation of HMGB1 at Lys^28,30,182–185^ by poly(ADP-ribose) polymerase-1 (PARP1) in response to inflammatory stimuli reduces its nuclear localization and promotes its translocation to the cytoplasm^39^. HMGB1 is glycosylated at Asn^37,134,135^, a modification that promotes its nucleocytoplasmic translocation and secretion^40^. Additionally, methylation at Lys^43^ has been identified as a regulatory modification that contributes to HMGB1 translocation and secretion in response to cellular stress^41^. The poly(ADP-ribosyl)ation of HMGB1 by PARP at its C-terminal acidic tail reduces its affinity for binding to DNA and its secretion. These modifications are induced during DNA damage and stress responses, which emphasizes HMGB1’s role as a DAMP in cellular stress and necrosis^42^. Ubiquitination of HMGB1 at its B-box by RING finger protein 186 (RNF186) and synoviolin 1 (SYVN1) leads to its degradation and reduces secretion. Conversely, deubiquitination by ubiquitin-specific peptidase (USP)12 and USP13, which directly bind to HMGB1, inhibits its ubiquitination. This prevents degradation and enhances stability, leading to increased cytoplasmic accumulation and extracellular release of HMGB1^43–46^. After undergoing various PTMs, HMGB1 translocates to the cytoplasm through chromosome region maintenance 1 (CRM1) and HSP90α family class A member 1 (HSP90AA1), where it is secreted via unconventional pathways and packaged into secretory autophagosomes and lysosomes^39,47^. Autophagy-mediated HMGB1 secretion involves diverse components of the autophagy machinery, vesicular trafficking systems and membrane fusion proteins, including Golgi reassembly stacking protein 2 (GORASP2), ADP ribosylation factor 1 (ARF1), secretion-associated Ras-related GTPase 1A (SAR1A) and Ras-related protein Rab11A/8/11/27A^47^ (Fig. 2c). These pathways are regulated so that HMGB1 functions as a DAMP to activate immune responses through diverse receptors.

## Intracellular role of HMGB1 oxidation

Accumulation of ROS inside cells under stressful conditions such as inflammation, infection or hyperglycemia induces redox-sensitive modifications in a variety of proteins, including HMGB1.

### HMGB1 oxidation mechanism

**Fig. 3:**
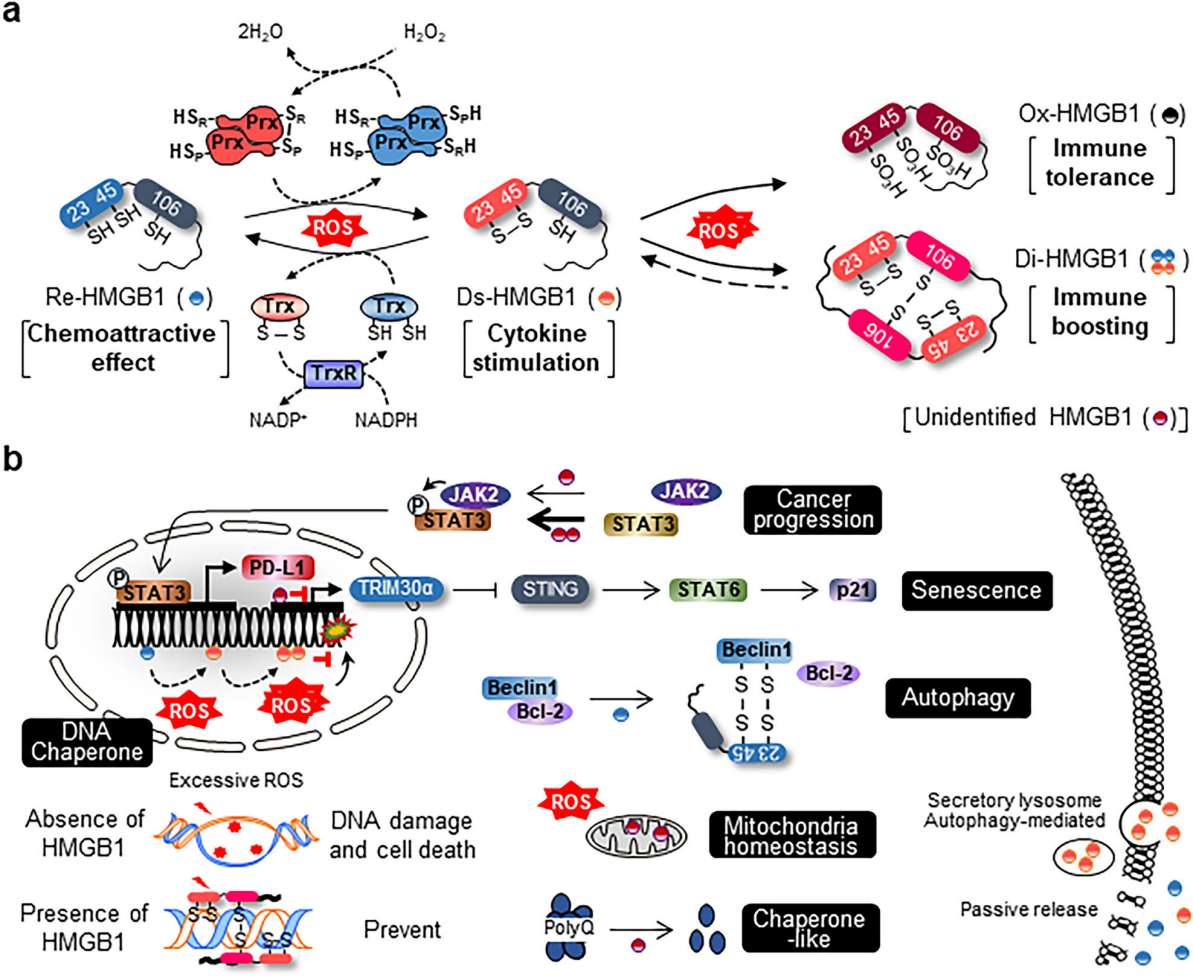
Redox regulation and intracellular function with resulting immune signaling of HMGB1. **a**, Schematic of redox-dependent transitions of HMGB1 in the nucleus. Under oxidative conditions, PrxI/II oxidize HMGB1 via thiol–disulfide exchange, generating Ds-HMGB1 (Cys^23^–Cys^45^). The Trx system (Trx/TrxR) restores Re-HMGB1. In the presence of excessive ROS, Cys^23^, Cys^45^ and Cys^106^ of HMGB1 are oxidized to sulfonic acid (–SO_3_H), leading to immune tolerance. Alternatively, the Cys^106^–Cys^106^ intermolecular disulfide bond forms Di-HMGB1 with enhanced proinflammatory properties. **b**, Cellular functions of HMGB1 redox isoforms. In the nucleus, HMGB1 acts as a DNA chaperone and regulates oxidative stress responses. Under excessive ROS, HMGB1 absence leads to DNA damage and cell death, while its presence promotes genome stability. HMGB1 modulates PD-L1 expression via the JAK2–STAT3 pathway and influences STING-mediated signaling through TRIM30α, promoting senescence (via p21) autophagy (Re-HMGB1 via Beclin-1–Bcl-2 complex disruption), and mitochondrial homeostasis. HMGB1 also exhibits chaperone-like activity in preventing PolyQ aggregation. Extracellular release occurs via secretory lysosomes, autophagy-mediated pathways or passive release.

One of the most prominent redox modifications of HMGB1 is the formation of an intramolecular disulfide bond between Cys^23^ and Cys^45^ (Ds-HMGB1). This process is tightly regulated by the antioxidant enzymes PrxI and PrxII. These enzymes contain catalytic thiol groups that react with ROS, such as hydrogen peroxide (H₂O₂). The peroxidatic cysteine in PrxI and PrxII oxidizes to sulfenic acid (Cys–SOH) when exposed to H₂O₂, which then reacts with a resolving cysteine to form an intra- or intermolecular disulfide bond. The oxidized PrxI and PrxII then transfer this oxidative signal to HMGB1 via thiol–disulfide exchange. This leads to the formation of a disulfide bond between HMGB1’s Cys^23^ and Cys^45^ (reaction: Prx–S–S + HMGB1–(SH)₂ → Prx–(SH)₂ + HMGB1–S–S)^32^. The Ds-HMGB1 isoform exhibits proinflammatory properties and preferentially activates TLR4 on immune cells. Concurrently, the Trx system can counterbalance this oxidation. Trx contains a conserved Cys–X–X–Cys motif that reduces Ds-HMGB1 through thiol–disulfide exchange (Trx–(SH)_2_ + HMGB1–S–S → Trx–S–S + HMGB1–(SH)₂), forming Re-HMGB1. Oxidized Trx is subsequently regenerated by Trx reductase (TrxR) using electrons from NADPH (Trx–S–S + NADPH + H⁺ → Trx–(SH)₂ + NADP⁺). Re-HMGB1 exhibits chemoattractive activity, particularly in recruiting immune cells under stress resolution conditions^48^. By contrast, under conditions of prolonged or excessive oxidative stress, HMGB1 undergoes irreversible hyperoxidation. Its cysteine residues progressively oxidize through sulfinic acid (–SO₂H) and sulfonic acid (–SO₃H) forms to create Ox-HMGB1. Ox-HMGB1 undergoes significant conformational changes resulting in the loss of DNA binding and receptor interactions. This renders the protein immunologically inert. This sulfonyl form of HMGB1 contributes to immune tolerance, particularly in late-stage inflammation or apoptotic contexts^49^. Interestingly, Cys^106^ of HMGB1 can also form an intermolecular disulfide bond, leading to dimerization of HMGB1 molecules (HMGB1–SH + HMGB1–SH → HMGB1–S–S–HMGB1), forming Di-HMGB1. Di-HMGB1 exhibits an enhanced proinflammatory capacity compared with the monomer. It shows stronger binding to receptors, such as TLR2 and TLR4, and potentiates cytokine release from immune cells^6^ (Fig. 3a). However, the precise mechanism remains undefined. These redox-driven modifications not only determine HMGB1’s localization (nuclear versus cytoplasmic) and secretion, but also critically modulate its immunological function. Depending on the oxidative environment, HMGB1 can transition from an immune-activating DAMP to an immunologically silent form.

### Function of intracellular HMGB1

In the nucleus, HMGB1 acts as a DNA chaperone that facilitates nucleosome remodeling and stabilizes chromatin structure. Under mild oxidative stress, ROS induce the formation of a disulfide bond between Cys^23^ and Cys^45^ via PrxI/II, generating the Ds-HMGB1 form^32^. Under conditions of excessive ROS accumulation, HMGB1 undergoes dimerization via an intermolecular disulfide bond between Cys^106^ and Cys^106^, resulting in an antiparallel dimer configuration. Di-HMGB1 strongly binds to DNA, preventing hydroxyl radical-induced DNA damage and enhancing cell survival^50^. In addition, nuclear HMGB1 contributes to cancer cell senescence by modulating the STING pathway through the suppression of TRIM30α, a negative regulator of STING. By downregulating TRIM30α expression, HMGB1 enhances STING signaling, leading to increased STAT6 phosphorylation and upregulation of p21^51^ (Fig. 3b).

HMGB1 also functions in the cytoplasm, where it plays a critical role in maintaining mitochondrial homeostasis. Multiple studies have demonstrated that cytoplasmic HMGB1 regulates mitochondrial quality control by modulating autophagy, mitophagy, mitochondrial dynamics and DNA repair mechanisms. Re-HMGB1 binds directly to Beclin-1, promoting the initiation of autophagy. This redox-sensitive interaction displaces Bcl-2 from Beclin-1, thereby facilitating the formation of autophagosomes and the clearance of dysfunctional mitochondria (mitophagy), a process essential for mitochondrial quality control and energy balance^52^. In addition, HMGB1 has been shown to promote mitochondrial fusion by downregulating dynamin-related protein 1 (Drp1) through the C–X–C chemokine receptor type 4 (CXCR4)–proteasome subunit β type-5 (PSMB5) axis, independently of the nuclear factor erythroid 2-related factor 2 (NRF2) pathway. This prevents excessive mitochondrial fragmentation and supports cellular homeostasis^53^. Cytoplasmic HMGB1 directly interacts with JAK2 and STAT3, enhancing JAK2–STAT3 complex formation and promoting STAT3 phosphorylation in breast cancer cells. This signaling cascade induces the expression of STAT3 target genes, including PD-L1, thereby facilitating tumor cell migration and invasion. Notably, Di-HMGB1 was found to more effectively potentiate JAK2–STAT3 activation than its monomeric HMGB1^54^. HMGB1 can also inhibit the aggregation of proteins such as polyglutamine (PolyQ), which induces neurodegenerative disorders. This indicates its chaperone-like activity^55^ (Fig. 3b). Therefore, investigating Di-HMGB1 in the nucleus and cytoplasm could provide critical insights into regulating cell survival and death and its pathophysiological relevance in cancer therapies. Cytoplasmic HMGB1 can be released either actively through unconventional secretion pathways involving secretory autophagosomes and lysosomes or passively as a result of cell death^14,39,47^.

## Extracellular role of HMGB1 oxidation

### HMGB1 oxidation mechanism

**Fig. 4:**
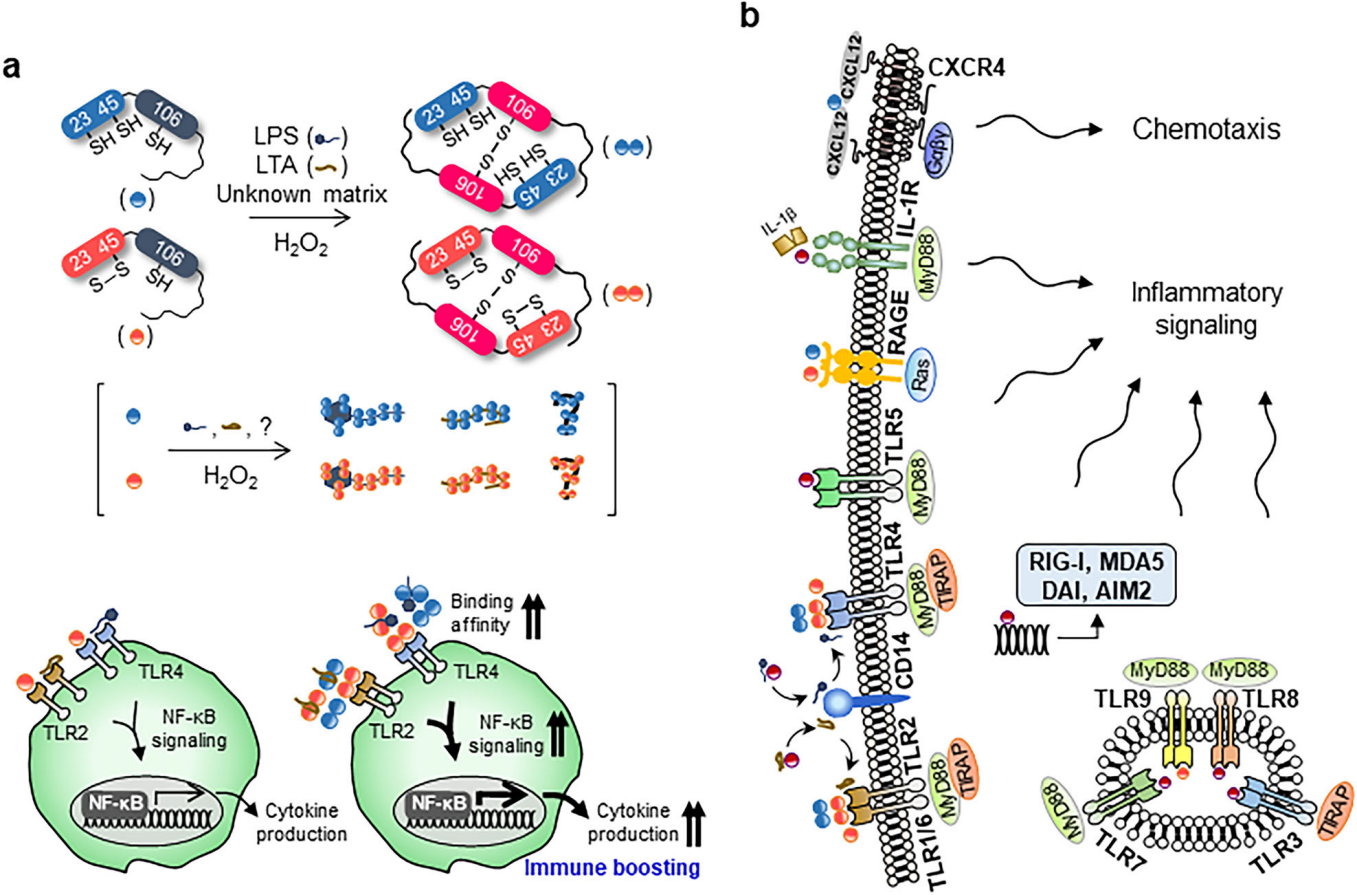
Extracellular redox regulation of HMGB1 and receptor-mediated immune activation. **a**, Redox state of HMGB1 under different conditions. In the extracellular space, H_2_O_2_ and bacterial components (LPS and LTA) promote oxidation and spatial proximity of HMGB1, leading to Cys^106^–Cys^106^ dimerization. Di-HMGB1 enhances binding affinity for TLR2 and TLR4, promoting NF-κB-dependent cytokine production and proinflammatory signaling. Redox-dependent dimerization functions as an immune-boosting mechanism during infection and inflammation. **b**, Extracellular HMGB1 interacts with multiple immune-regulating receptors: TLRs, RAGE, IL-1R and CXCR4. These interactions activate downstream signaling pathways that trigger chemotaxis and inflammatory responses. Cytosolic DNA sensors RIG-I, MDA5, DAI and AIM2 also respond to HMGB1-associated stimuli.

Both the reduced and oxidized forms of HMGB1 are present in the extracellular space. LPS and LTA are well-characterized PAMPs that initiate immune responses. These molecules act as binding platforms that promote extracellular HMGB1 dimerization, a process primarily driven by ROS-dependent oxidation of Cys^106^. ROS selectively oxidize the thiol group of Cys^106^ in HMGB1. First, it is converted to sulfenic acid (–SOH), which then facilitates disulfide bond formation between two HMGB1 molecules at this site: HMGB1–SH + HMGB1–SH + ROS → HMGB1–S–S–HMGB1. LPS and LTA promote the spatial proximity of HMGB1 molecules in the extracellular environment. The resulting HMGB1 dimer demonstrates an enhanced binding affinity for TLR2 and TLR4, which sustains NF-κB activation and drives robust proinflammatory cytokine release^6^ (Fig. 4a).

### Function of extracellular HMGB1

Depending on its redox state, HMGB1 interacts with distinct receptors to exert its function as a DAMP, contributing to immune responses by promoting chemotaxis or stimulating the release of proinflammatory cytokines (Fig. 4b). HMGB1 facilitates proinflammatory signaling by interacting with TLR2. Di-HMGB1 binds more strongly to TLR2 than monomeric HMGB1, further enhancing NF-κB signaling^6^. HMGB1 has been shown to interact with LTA and facilitate its transfer to CD14 and TLR2. This amplifies the production of proinflammatory cytokines, such as tumor necrosis factor (TNF-α) and IL-6^56^. Ds-HMGB1 has also been shown to negatively affect the maturation of oligodendrocyte progenitor cells via TLR2 signaling^57^. Yang et al. demonstrated that Ds-HMGB1, but not Re- or Ox-HMGB1, directly binds to MD2/TLR4 and induces the release of the proinflammatory cytokine TNF-α^58^. In addition, HMGB1 binds to LPS, promoting its transfer to CD14 and TLR4 and augmenting LPS-induced TNF-α production in human monocytes^30^. Synthetic peptides HPep1 and HPep6, derived from the A box and B box domains of HMGB1, block LPS transfer to CD14, reduce cellular uptake in RAW264.7 cells and suppress LPS-induced TNF-α production in human peripheral blood mononuclear cells and a mouse endotoxemia model^31^. Di-HMGB1 has a higher binding affinity to TLR4 than monomeric HMGB1 and markedly enhances NF-κB signaling^6^. A pivotal study by Das et al. elucidated that extracellular HMGB1 binds to TLR5, initiating a proinflammatory signaling cascade via the MyD88-dependent pathway. The C-terminal acidic tail of HMGB1 plays a crucial role in facilitating this binding to TLR5^59^. Both Re- and Ds-HMGB1 have been shown to interact directly with RAGE. Hepatocyte-derived Ds-HMGB1 interacts with RAGE to activate proinflammatory signaling in alcohol-associated liver disease^60^. Extracellular Re-HMGB1 enhances the senescence-associated secretory phenotype (SASP), which includes IL-6, IL-8 and TNF-α, via RAGE-mediated NF-κB signaling^61^. In addition, the HMGB1–IL-1β complex has been shown to strongly enhance NF-κB signaling through the activation of extracellular signal-regulated kinase (ERK)1/2 and p38 mitogen-activated protein kinase pathways^62^. Re-HMGB1 forms a heterocomplex with the C–X–C motif chemokine ligand 12 (CXCL12), which subsequently binds to the CXCR4, enhancing chemotactic signaling and promoting inflammatory cell recruitment^63^. Yanai et al.’s study elucidates the role of HMGB proteins, including HMGB1, as universal sentinels in nucleic acid-mediated innate immune responses. HMGB1 binds to various nucleic acids, such as DNA and RNA, and facilitates their recognition by TLRs, including TLR3, TLR7 and TLR9. This binding enhances the activation of downstream signaling pathways, leading to the production of type I interferons and proinflammatory cytokines^64^. Re-HMGB1 has also been shown to modulate TLR3 signaling pathways. In keratinocytes, pretreatment with Re-HMGB1 attenuates polyinosinic:polycytidylic acid [poly(I:C)]-induced activation of TLR3 signaling^65^. Sosa et al. investigated the immunological factors contributing to varied liver transplant outcomes among female Hispanic patients with non-alcoholic steatohepatitis. The study identified elevated HMGB1 levels and increased TLR4, TLR8 and TLR9 activation in this patient population^66^. Ds-HMGB1 has been identified as the variant that promotes TLR9-mediated inflammatory responses. Ds-HMGB1 engages both the TLR4 and TLR9 pathways, leading to the polarization of macrophages toward a proinflammatory phenotype^67^. HMGB1 in complex with immunogenic DNA or RNA is recognized by cytosolic sensors such as RIG-I, melanoma differentiation-associated gene 5 (MDA5), DNA-dependent activator of interferon regulatory factors (DAI) and absent in melanoma 2 (AIM2), leading to the activation of innate immune responses^64^ (Fig. 4b). Conversely, the interaction between HMGB1 and its receptors has also been implicated in anti-inflammatory signaling. Specifically, its interaction with TIM-3 on dendritic cells (DCs) inhibits the endosomal accumulation of nucleic acids, thereby reducing the immunogenicity of nucleic acids released from dying tumor cells. This mechanism has been associated with diminished efficacy of DNA vaccination and chemotherapy^68^. Furthermore, both LPS and LTA have been shown to induce intermolecular dimerization of HMGB1. The resulting LPS–Di-HMGB1 and LTA–Di-HMGB1 complexes exhibited markedly enhanced proinflammatory activity compared with their respective monomeric counterparts, LPS–HMGB1 and LTA–HMGB1. Notably, these dimeric complexes more effectively amplified TLR4- and TLR2-mediated signaling, respectively, resulting in elevated downstream inflammatory cytokine production^6^. These findings suggest that dimerization of HMGB1 may induce conformational rearrangements that enhance its affinity for bacterial and potentially viral components such as RNA and DNA molecules, thereby facilitating a more stable and efficient transfer of these ligands to their corresponding receptors. This implies that HMGB1 dimerization serves a structural mechanism that potentiates PAMP recognition and amplifies innate immune signaling cascades under oxidative or inflammatory conditions. The redox states of HMGB1 play distinct and critical roles in modulating inflammatory signaling pathways.

## HMGB1 and cell death

Both Re- and Ds-HMGB1 can be passively released during cell death. Once extracellular, these redox isoforms exert distinct functions, promoting either survival or further cell death. This section examines the mechanisms underlying HMGB1 release and how its redox-dependent functions affect cell fate.

### Release of various oxidized forms of HMGB1 by cell death

**Fig. 5:**
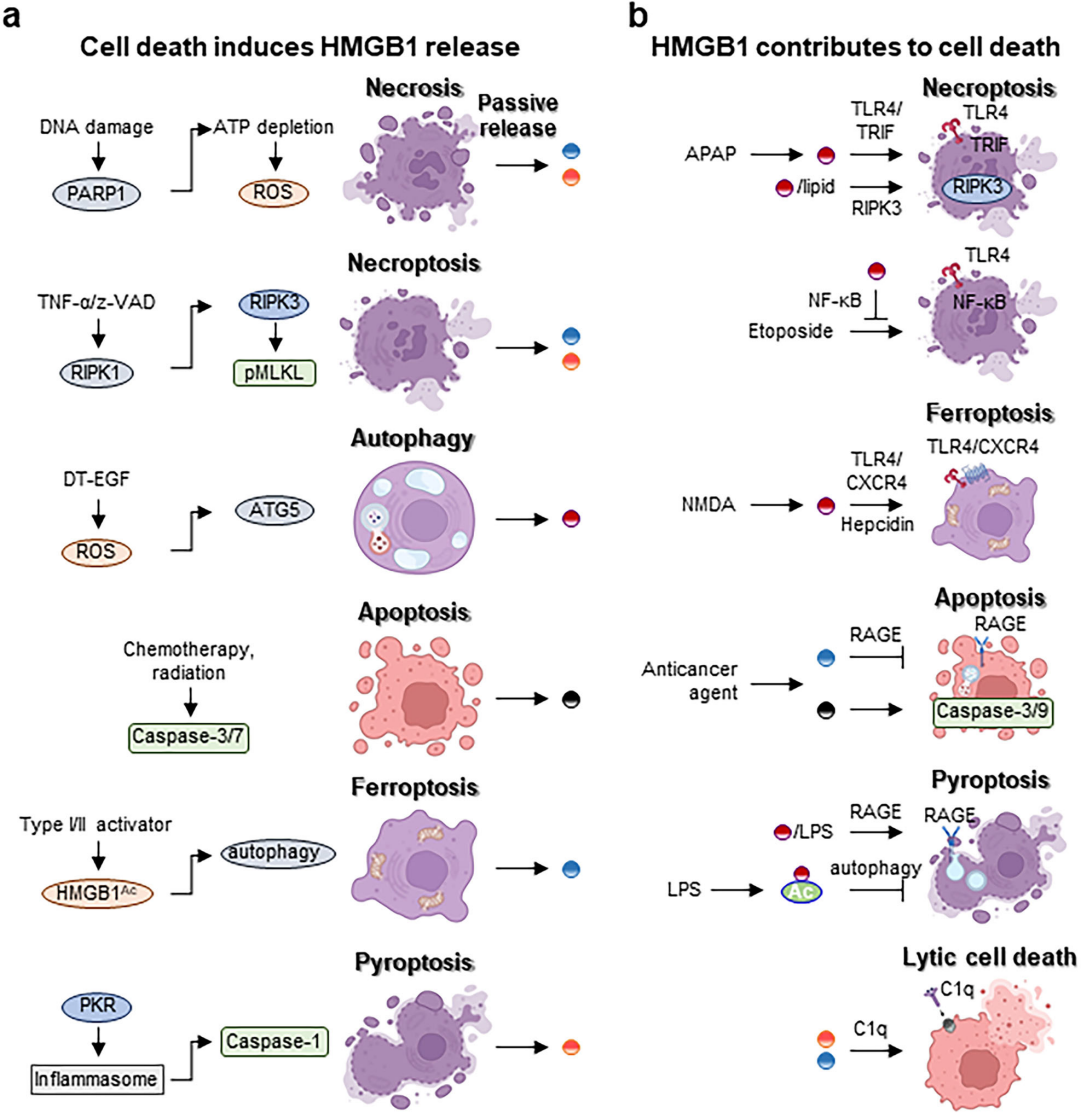
Bidirectional relationship between HMGB1 and regulated cell death. **a**, Multiple forms of regulated cell death trigger passive release of HMGB1 into the extracellular space. Each cell death involves distinct upstream signals such as PARP1 activation, caspase cleavage, autophagy induction or inflammasome activation. The redox state of released HMGB1 varies according to the type of cell death and oxidative context. **b**, Released HMGB1 modulates various cell death pathways in an autocrine or paracrine manner. HMGB1 amplifies necroptosis via TLR4/TRIF-mediated signaling pathway, and HMGB1–lipid complex engages RIPK3 phosphorylation to promote necroptosis. HMGB1 activates ferroptosis through hepcidin upregulation via TLR4–CXCR4 signaling. Ox-HMGB1 facilitates apoptosis through caspase-3/9 activation, while Re-HMGB1 promotes Beclin-1-dependent autophagy via RAGE. HMGB1 drives pyroptosis through RAGE-mediated endocytosis and caspase-1 activation. In lytic death, both Re- and Ds-HMGB1 activate C1q and complement-mediated MAC deposition. HMGB1^AC^, HMGB1 acetylation.

Necrosis is triggered by stimuli such as hypoxia, DNA damage or alkylating agents. DNA damage activates PARP1, which facilitates the translocation of HMGB1 from the nucleus to the cytoplasm prior to cell death (Fig. 5a). PARP1-deficient cells do not exhibit HMGB1 translocation following DNA damage^42^. Early in necrosis, HMGB1 is primarily released in its reduced form. As necrosis progresses, rising ROS levels oxidize HMGB1^69^. Necroptosis is initiated by signals such as TNF-α in the presence of caspase inhibitors, including z-VAD-fmk. This triggers the phosphorylation of receptor-interacting serine/threonine kinase (RIPK)1/RIPK3 and the activation of mixed-lineage kinase domain-like pseudokinase (MLKL), resulting in HMGB1 translocation and release^70^. Mitochondria generate ROS when disrupted during necroptosis and further oxidize HMGB1^71^. Autophagic cell death, which is often induced by toxins such as diphtheria toxin-EGF in glioblastoma cells, can lead to HMGB1 release without compromising membrane integrity. Knockdown of autophagy genes (*ATG5*, *ATG7* and *ATG12*) inhibits HMGB1 release despite cell death; however, the oxidation state of HMGB1 was not specified^72^. Apoptosis is generally considered immunologically silent. HMGB1 remains sequestered in apoptotic vesicles, preventing its release into the extracellular release^73^. In addition, apoptosis-associated ROS generation results in HMGB1 hyperoxidation, rendering it immunologically inactive^49^. Ferroptosis, a form of cell death dependent on iron and lipid peroxidation, is induced by type I and type II activators, such as erastin and RSL3, respectively. These inducers promote HMGB1 acetylation and autophagy-mediated release. Deletion of autophagy genes or the use of inhibitors prevents the release of acetylated HMGB1^74^. Pyroptosis is driven by inflammasome-activated caspase-1/11 and gasdermin-mediated pore formation. This process leads to the release of HMGB1 alongside IL-1β and IL-18. Protein kinase R (PKR) is essential for inflammasome activation^75^. Ds-HMGB1 is the predominant form released during pyroptosis^76,77^ (Fig. 5a). PANoptosis is a combined form of pyroptosis, apoptosis and necroptosis triggered during severe acute respiratory syndrome coronavirus 2 (SARS-CoV-2) infection. It results in the release of multiple HMGB1 PTM forms, including Ds-HMGB1^78^. Extracellular HMGB1 binds directly to the SARS-CoV-2 spike protein, causing RAGE-mediated infection by endocytosis^79^.

### HMGB1 induces diverse cell death

Under certain extracellular conditions or in specific cell types, HMGB1 also actively contributes to the induction of various forms of cell death (Fig. 5b). Acetaminophen (*N*-acetyl-*para*-aminophenol, APAP) treatment leads to the release of HMGB1 from necrotic hepatocytes. Extracellular HMGB1 then acts in a feed-forward manner to exacerbate hepatocyte necrosis. In HepaRG cells, HMGB1 induces cell death via TLR4 and its adaptor protein, TIR-domain-containing adapter-inducing interferon-β (TRIF), leading to the activation of RIPK3^80^. In addition, HMGB1 released from damaged or necrotic cells indirectly amplifies injury by recruiting neutrophils. These neutrophils exacerbate tissue damage and necrosis, establishing a vicious cycle of injury amplification^81^. Extracellular HMGB1 forms complexes with bacterial lipid components, such as lipid IVa and lipid A, which are recognized and activated through TLR4–TRIF–RIPK3 signaling pathways during MLKL-mediated necroptosis^82^. Conversely, HMGB1 prevents necroptosis via NF-κB activation in acute myeloid leukemia cells treated with etoposide and z-VAD-fmk, suggesting context-dependent outcomes^83^. In cerebral ischemia, *N*-methyl-*D*-aspartate treatment induces HMGB1 secretion, and extracellular HMGB1 activates ferroptosis in astrocytes through TLR4–CXCR4 signaling and hepcidin upregulation. This induces intracellular iron overload, lipid peroxidation and ferroptosis, contributing to neuronal injury^84^. The redox form of HMGB1 determines whether it induces apoptosis or autophagy in cancer cells. Anticancer agents such as melphalan and paclitaxel induce the secretion of Re-HMGB1 and Ox-HMGB1, and Re-HMGB1 binds to RAGE and stimulates Beclin-1-dependent autophagy to promote cell survival. However, Ox-HMGB1 induces intrinsic apoptosis via caspase-3/9 pathways, thereby enhancing treatment-induced cytotoxicity^85^. HMGB1 can either promote or suppress pyroptosis. When HMGB1 binds to LPS, the complex is internalized via a RAGE- and dynamin-dependent pathway. This disrupts lysosomal membranes, allowing LPS to escape into the cytoplasm and activate the noncanonical inflammasome via caspase-11, inducing pyroptosis^86^. In LPS-stimulated RAW264.7 macrophages, cytoplasmic acetylated HMGB1 promotes early autophagy, whereas extracellular HMGB1 induces late-stage pyroptosis^87^. Both Re- and Ds-HMGB1 forms bind to complement component C1q, activating the antibody-independent classical complement cascade and ultimately forming C3a and C5b-9 membrane attack complexes (MACs). These complexes contribute to sterile inflammation and further cell death^88^ (Fig. 5b). Re-HMGB1 induces senescence in fibroblasts through the RAGE-dependent NF-κB and JAK–STAT signaling pathways^61^. Although the redox states of HMGB1 released during different cell death processes are increasingly well defined, the specific redox isoform that induces subsequent cell death is not fully characterized.

### HMGB1 balances between cell death and survival

HMGB1 serves as a critical regulator of cell fate, modulating the balance between survival and regulated cell death. Its function is tightly controlled by its redox state, subcellular localization and receptor interactions such as RAGE and TLRs. Under cellular stress, nuclear Re-HMGB1 translocates to the cytoplasm, where it binds to Beclin-1, disrupting the Beclin-1–Bcl-2 complex. This process promotes autophagy, a protective mechanism that preserves mitochondrial integrity, prevents apoptosis and contributes to therapy resistance in cancer cells^89^. In addition, extracellular Re-HMGB1 induces Beclin1-dependent autophagy through RAGE binding, whereas Ox-HMGB1 can promote apoptosis via caspase3/9 intrinsic pathway. Under certain chemotherapeutic conditions, cytosolic HMGB1 shifts from promoting autophagy to directly activating apoptosis when autophagic pathways are overwhelmed^85^. In macrophages, HMGB1 initially promotes autophagy in response to LPS stimulation. However, sustained or excessive stimulation results in the extracellular release of HMGB1, promoting caspase-1-mediated pyroptosis^87^. HMGB1 also plays a pivotal role in modulating the balance between cellular senescence and apoptosis. In B16 mouse melanoma cells treated with doxorubicin, knocking out HMGB1 led to enhanced apoptosis accompanied by decreased p21 expression. Conversely, HMGB1 overexpression shifted the cellular response from apoptosis to senescence, which correlated with increased p21 levels^90^. Thus, depending on cellular context, timing and its redox states, HMGB1 functions as either a survival factor or an inflammatory cell-death trigger.

## HMGB1 oxidation and immune disorders

**Fig. 6:**
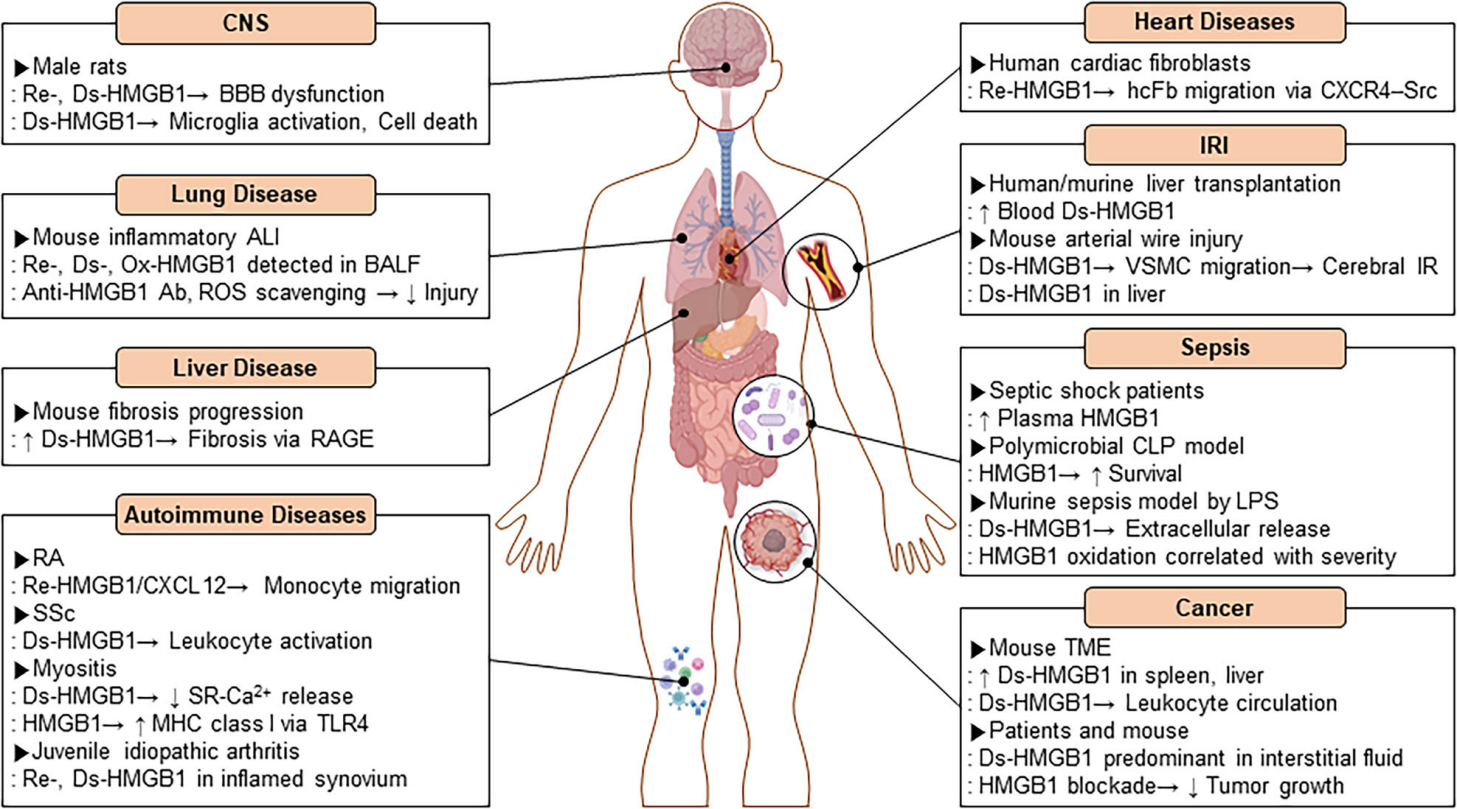
Disease-specific distribution of HMGB1 redox isoforms in human and experimental models. A schematic of distinct HMGB1 redox forms in various pathological contexts. The redox status of HMGB1 varies spatially and temporally across diseases, modulating immune responses and tissue pathology. CNS, central nervous system; BBB, blood–brain barrier; ALI, acute lung injury; BALF, bronchoalveolar lavage fluid; RA, rheumatoid arthritis; SSc, systemic sclerosis; SR-Ca^2+^, sarcoplasmic reticulum Ca^2+^; hcFb, human cardiac fibroblast; VSMC, vascular smooth muscle cell.

The redox state of HMGB1 is associated with time and tissues in certain pathological conditions^91^. Multiple studies in animal models and clinical settings have demonstrated that specific HMGB1 redox states contribute to disease pathogenesis (Fig. 6). Intracortical injection of Re- and Ds-HMGB1 induces blood–brain barrier disruption in rats, and Ds-HMGB1 increases microglia activation and apoptosis in the central nervous system^92^. Elevated HMGB1 levels have been observed in traumatic brain injury^93^, epilepsy^94^, Alzheimer’s disease^95^ and Parkinson’s disease^96^. Maria Entezari et al. reported that, in hyperoxic lung injury, all redox forms of HMGB1 are present in bronchoalveolar lavage fluid, with Re-HMGB1 predominating extracellularly. Inhibiting HMGB1 secretion or neutralizing anti-HMGB1 antibody mitigates hyperoxia-induced acute lung injury^97^. In carbon tetrachloride-induced liver fibrosis, Ds-HMGB1 levels increase during injury, whereas Ox-HMGB1 levels increase during resolution. Only Ds-HMGB1 drives fibrogenesis via RAGE. In severe acute pancreatitis, Ds-HMGB1 correlates with severe inflammation, gut barrier dysfunction, bacterial translocation and organ damage^98^. Redox regulation of HMGB1 plays an important role in autoimmune diseases. In rheumatoid arthritis, for example, elevated Trx/Trx reductase (TrxR) maintains HMGB1 in its reduced form, thereby enhancing monocyte migration via CXCL12. Blocking Trx or JAK2/COX2 increases HMGB1 oxidation and reduces inflammation^99^. In addition, oxidative stress induces Ds-HMGB1 from platelets, activating neutrophils via TLR4-driven ROS production and autophagy in systemic sclerosis^100^. In inflammatory myositis, only Ds-HMGB1, not Re-HMGB1, impairs calcium signaling via TLR4 and induces major histocompatibility complex (MHC) I upregulation in muscle fibers^101^. Lundbäck et al. demonstrate that Re- and Ds-HMGB1 are prevalent in the inflamed synovial fluid of patients with juvenile idiopathic arthritis^102^. HMGB1 has also been associated with cardiac dysfunction. In cardiac fibroblasts, the Re- or ‘3S’ non-oxidizable HMGB1 mutant induces migration via CXCR4 interaction and proto-oncogene tyrosine-protein kinase Src activation, unlike Ds-HMGB1^103^. In human liver transplantation, elevated levels of Ds-HMGB1 in portal blood correlate with the severity of ischemia–reperfusion injury (IRI) and drive TNF-α release via TLR4^104^. In murine models, HMGB1 neutralization reduces TLR4-dependent injury in renal and cerebral IRI^105,106^ and Ds-HMGB1 induces smooth muscle migration via TLR4–MyD88/NF-κB, promoting intimal hyperplasia^107^. Following cerebral IRI, Ds-HMGB1 stimulates hepcidin via TLR4/RAGE in the liver, causing iron dysregulation and damage^108^. HMGB1 is one of the major inflammatory mediators of sepsis, along with TNF-α, IL-1, IL-6 and IL-8. In septic shock patients, high circulating HMGB1 levels correlate with disease severity, proinflammatory cytokine balance and mortality. Anti-HMGB1 treatment improves survival in models of cecal ligation and puncture^109^. In LPS-induced murine sepsis, HMGB1 oxidation mirrors disease severity and correlates with impaired mitochondrial function and increased cytokine release^110^. HMGB1 redox isoforms play context-dependent roles in cancer. Ds-HMGB1 is closely associated with proinflammatory signaling and promotes tumor growth by encouraging cancer cell proliferation, migration and metastasis. In the TME, Ds-HMGB1 is predominantly enriched in the spleen and liver, whereas its levels are markedly reduced in cachectic skeletal muscle tissues. In addition, Ds-HMGB1 induces leukocyte circulation and infiltration and is the predominant isoform in the interstitial fluids of the TME^91^. Blocking HMGB1 drastically reduces tumor growth by decreasing monocytic/granulocytic myeloid-derived suppressor cells and regulatory T lymphocytes. This increases the M1/M2 macrophage ratio and activates DCs and plasmacytoid DCs in the TME^111^ (Fig. 6). Moreover, Di-HMGB1 can be detected not only in the serum but also in the liver tissue of mice following intraperitoneal injection of LPS, as well as in subcutaneous tumor tissues exposed to ultraviolet irradiation^50^. These findings suggest that Di-HMGB1 formation is not confined to a single tissue type but rather occurs in various organs under conditions of elevated ROS. Given its potent proinflammatory activity, Di-HMGB1 may also contribute to the pathogenesis of immune disorders associated with excessive or chronic inflammation. These findings support the possibility that oxidative stress associated with infection or cellular injury can promote Di-HMGB1 generation across multiple tissues.

## Concluding remarks and perspectives

HMGB1 is a prototypical DAMP that plays a critical role in inflammation and immune regulation. Its biological functions are governed by subcellular localization and redox status. HMGB1 contains three redox-sensitive cysteine residues that undergo specific oxidative modifications, which enable distinct functional roles, such as maintaining genomic integrity in the nucleus, regulating autophagy and mitochondrial homeostasis in the cytoplasm, and modulating immune responses in the extracellular environment. Re-HMGB1 predominantly facilitates tissue repair via CXCR4 signaling. By contrast, Ds-HMGB1 activates TLRs and RAGE, driving proinflammatory cytokine release. Furthermore, HMGB1 release mode and its redox state are tightly coupled to distinct forms of cell death, including necrosis, necroptosis, autophagy, apoptosis, ferroptosis and pyroptosis. These redox transitions determine the immunological fate of the dying cell and shape the surrounding inflammatory milieu. Pathophysiologically, redox-specific HMGB1 isoforms play different roles in various immune-related disorders. Ds-HMGB1 is associated with chronic inflammation, cytokine storms and disease exacerbation in conditions such as sepsis, autoimmune diseases, neuroinflammation and cancer. This emerging HMGB1 dimerization mechanism represents a poorly explored yet potentially significant mode of redox-dependent immunoregulation, particularly in oxidative microenvironments associated with infection and tissue injury. In vitro experiments demonstrated that an increase in ROS levels induces conformational changes of Re-HMGB1 to Ds-HMGB1 and Di-HMGB1. Furthermore, in vivo studies confirmed the presence of both Ds-HMGB1 and Di-HMGB1 in ROS-inducing conditions, such as LPS injection or ultraviolet irradiation. However, the oxidation threshold of Ds-HMGB1 and Di-HMGB1 in the parenchyma or interstitial fluid across time and space in pathological conditions remains uncharacterized. The variation in HMGB1 oxidation states in specific tissues and conditions is likely to provide crucial insights into the underlying mechanisms of HMGB1-mediated inflammation.
